# A Novel Approach for Studying the Physiology and Pathophysiology of Myelinated and Non-Myelinated Axons in the CNS White Matter

**DOI:** 10.1371/journal.pone.0165637

**Published:** 2016-11-09

**Authors:** Lijun Li, Alexander A. Velumian, Marina Samoilova, Michael G. Fehlings

**Affiliations:** 1 Division of Genetics and Development, Krembil Research Institute, University Health Network, Toronto, Canada; 2 Division of Neurosurgery, University Health Network, Toronto, Canada; 3 Krembil Neuroscience Center, University Health Network, Toronto, Canada; 4 Department of Physiology, Faculty of Medicine, University of Toronto, Toronto, Canada; 5 Department of Surgery, Faculty of Medicine, University of Toronto, Toronto, Canada; Emory University, UNITED STATES

## Abstract

Advances in brain connectomics set the need for detailed knowledge of functional properties of myelinated and non-myelinated (if present) axons in specific white matter pathways. The corpus callosum (CC), a major white matter structure interconnecting brain hemispheres, is extensively used for studying CNS axonal function. Unlike another widely used CNS white matter preparation, the optic nerve where all axons are myelinated, the CC contains also a large population of non-myelinated axons, making it particularly useful for studying both types of axons. Electrophysiological studies of optic nerve use suction electrodes on nerve ends to stimulate and record compound action potentials (CAPs) that adequately represent its axonal population, whereas CC studies use microelectrodes (MEs), recording from a limited area within the CC. Here we introduce a novel robust isolated "whole" CC preparation comparable to optic nerve. Unlike ME recordings where the CC CAP peaks representing myelinated and non-myelinated axons vary broadly in size, "whole" CC CAPs show stable reproducible ratios of these two main peaks, and also reveal a third peak, suggesting a distinct group of smaller caliber non-myelinated axons. We provide detailed characterization of "whole" CC CAPs and conduction velocities of myelinated and non-myelinated axons along the rostro-caudal axis of CC body and show advantages of this preparation for comparing axonal function in wild type and dysmyelinated *shiverer* mice, studying the effects of temperature dependence, bath-applied drugs and ischemia modeled by oxygen-glucose deprivation. Due to the isolation from gray matter, our approach allows for studying CC axonal function without possible "contamination" by reverberating signals from gray matter. Our analysis of "whole" CC CAPs revealed higher complexity of myelinated and non-myelinated axonal populations, not noticed earlier. This preparation may have a broad range of applications as a robust model for studying myelinated and non-myelinated axons of the CNS in various experimental models.

## Introduction

White matter comprises nearly half of brain volume and contains important axonal pathways interconnecting different parts of the brain. Detailed understanding of functional properties of cerebral white matter tracts is important for diagnosis and treatment of a variety of brain disorders including stroke, traumatic brain injury, demyelinating and neuroinflammatory conditions, brain tumors, neurodegenerative conditions, neonatal hypoxic-ischemic encephalopathy and stroke. Recent advances in brain connectomics [1–7] provided detailed knowledge on structural framework of brain wiring, while the functional properties of axons in many white matter pathways remain less explored. This particularly applies to conduction properties of axons that define the precise timing of signal delivery that is important for coordinated activity of CNS networks.

The corpus callosum (CC) is a major white matter commissural structure mediating interhemispheric signaling in placental animals and humans [8,9]. The rat and mouse CC has been extensively used for electrophysiological and pharmacological studies of axonal function and dysfunction in the CNS [10–17], specifically for studying the myelination in development [18–20], demyelination/remyelination in adulthood [15,21–26], effects of anoxia/ ischemia [27–31] and traumatic brain injury [13,14,16,32,33]. An important advantage of studying the CC is that, unlike another widely studied white matter structure, the optic nerve [34–40], where virtually all axons are myelinated, the CC contains not only myelinated but also a large population of non-myelinated axons [10,27,41].

The axonal function in CC had been traditionally studied by recording compound action potentials (CAPs) using extracellular microelectrodes (MEs) *in vivo* [10] or *in vitro* in brain slices [11,13–17,42]. ME recordings within CC reflect only a limited axonal population around the ME tip, recorded at short, 1–2 mm distance from stimulation site. Furthermore, CC CAPs recorded *in vivo* or in slices may be complicated by reverberating signals from the cortex, as emphasized in [10]. Two more recent studies using ME recordings in rat coronal brain slices [11,14] have attempted to rule out reverberations by lowering the Ca^2+^ concentrations in the bathing media, however because Ca^2+^ was lowered to 0.5 mM but not completely removed, the block of synaptic transmission may have been incomplete. Furthermore, the rostro-caudal position of the slices where these tests were done was not identified in these papers, leaving uncertainties about the presence of the whole path to cortex within the tested slices. Ruling out reverberating signals requires a complete isolation of CC from other structures. We therefore developed an isolated *in vitro* "whole" CC preparation dissected from mouse brain slices, allowing for suction electrode (SE) recordings from larger axonal populations and at longer distances for a better separation of CAP components, which are not achievable by ME recording.

This paper demonstrates the advantages of isolated “nerve-like” CC strips ("whole" CC), dissected from mouse brain slices, as a robust viable preparation allowing for improved electrophysiological characterization of axonal function and studying the effects of drugs and modeled ischemia on myelinated and non-myelinated axons of CNS white matter. Of particular importance is the applicability of this novel approach to studying the effects of demyelination, as exemplified by using dysmyelinated *shiverer* mouse CC, uncovering features not detected in earlier ME studies.

Our novel robust approach for studying the physiology and pathophysiology of myelinated and non-myelinated axons is applicable not only to CC but to other white matter structures, adding a functional component to brain connectivity data that are now available through several fundamental projects [1,4,43].

## Materials and Methods

### Mice

The c57bl/6 (wild type; wt) and myelin-deficient mutant *shiverer* (shi^-/-^) mice were obtained from Harlan laboratories, the latter being bred locally at the animal facility. All experimental protocols were approved by the Animal Care Committee of the University Health Network in accordance with the guidelines of Canadian Council of Animal Care. A total of 29 wt and 11 shi^-/-^ mice, 2.5–3.5 months-old, were used.

### Mouse brain slices and the "whole" CC dissection

Mice were deeply anesthetized with sodium pentobarbital (60 μg/g, i.p.) and transcardially perfused with oxygenated ice-cold sucrose-substituted artificial cerebrospinal fluid (aCSF, see Solutions) before decapitation and dissection of the brain. Seven serial 400 μm-thick coronal slices of the brain containing the CC were obtained on Leica vibrating microtome VT1200S. For extracellular microelectrode recording within the slices, the slices were incubated in oxygenated (95%O_2_ + 5% CO_2_) aCSF at room temperature for at least an hour before use.

**Fig 1:**
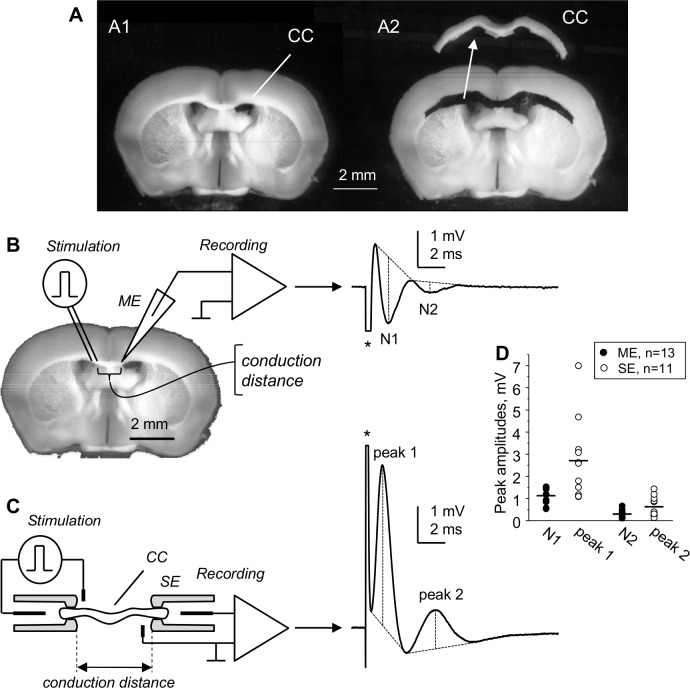
The isolated corpus callosum (CC) for suction electrode (SE) recording of compound action potentials (CAPs). (A) 400 μm-thick coronal slice of mouse brain before (A1) and after (A2) dissecting out the CC. The isolated CC is shown in A2 above the slice. (B) Field microelectrode (ME) recording of CC CAPs within brain slices, showing two negative peaks conventionally referred to as N1 and N2. (C) Our novel “whole” CC recording of CAPs from isolated CC using SE stimulation and recording, showing two positive peaks 1 and 2. The CAPs in B and C were maximal and were averaged from 6 consecutive traces taken at 10 s intervals. Stimulus artifacts are marked with (*) in (B) and (C). Dashed lines on CAPs explain the measurements of peak amplitudes from their projected bases. (D) Amplitudes of CAP peaks recorded at 1.5–2 mm conduction distances in different slices using ME (black circles) or in isolated CCs using SE (open circles) at room temperature. Horizontal bars in (D) indicate mean amplitudes of ME and SE recorded CAP peaks. Note larger amplitudes of SE-recorded “whole” CC CAP peaks compared to ME recordings. The “whole” CC SE recording more adequately characterizes the axonal population of CC spanning between the stimulated and recorded sites compared to ME recording.

For the "whole" CC recording, which is a novel approach developed by us, the CCs were dissected under Leica MS5 stereomicroscope from 400 μm-thick coronal slices in oxygenated ice-cold sucrose-substituted aCSF within 20–30 minutes after slicing. As shown in S1 Video, we used the sharp edge of a hypodermic needle as an improvised microscalpel. Fig 1A shows a slice before and after dissecting out the "whole" CC. The slice shown in Fig 1A2 was preserved during dissection for illustration purposes. The images and videos of dissection were taken using Leica IC80 HD camera mounted on the stereomicroscope. Without preserving the rest of slice tissue and with minimal practice, a dissection of "whole" CC from a brain slice takes 3–4 minutes.

The dissected "whole" CCs were incubated at room temperature in identifiable positions on a mesh in oxygenated aCSF, similarly to brain slices for at least one hour before use, and remained viable for at least 6–8 hours as tested randomly by electrophysiological recordings.

### Compound action potential (CAP) recording and data analysis

The recordings were performed in a 0.5 ml bath continuously perfused at 1 ml/min rate with a standard aCSF or switched to test solutions for studying the effects of potassium channel blockers or oxygen-glucose deprivation (OGD) (see Solutions).

Extracellular field ME recordings (Fig 1B) were performed in coronal brain slices using 3M NaCl-filled MEs with 2–3 μm tip diameters as described earlier [26]. "Whole" CC CAPs were recorded with SEs attached to opposite ends of the preparation (Fig 1C).

For SE recording and stimulation, the ends of isolated CC were drawn into SEs under visual control under a Leica WILD M3Z stereomicroscope. The SEs, with inner diameters of "mouths" around 300 μm, were inserted horizontally through the opposite sides of the bath and positioned at a desired distance between the tips (2 or 4 mm). This allowed for covering the bath with a plastic hood during recordings, thus reducing the gas loss from the perfusing solutions. The suction electrodes were connected to amplifier via an inserted Ag/AgCl wire for recording, and connected to inter-switchable 1 ml and 10 ml syringes via 3-way connectors for applying negative ("suction") pressure. The second electrodes in the pairs for recording and stimulation were Ag/AgCl wires inserted through the side walls of the bath, with their ends "wrapped" around the SEs, ~1 mm back from their tips (S1 Fig). The position of each CC within the SEs was photographed through the stereomicroscope using a home-made webcam attachment to the objective, and the distances along CCs between the "mouths" of stimulating and recording SEs ("conduction distances") were verified for each recording. The procedure of drawing the ends of CCs into the SEs included an initial "suction" using 1 ml syringe, applied for periods of 20–40 s in duration and 0.5–1 psi (~25–50 mm Hg) in magnitude (measured in selected experiments using a DWYER Series 475 Mark III digital manometer connected in-line with 1 ml syringe used for applying the negative pressure). After that, the negative pressure was lowered to a "holding" level by switching to a 10 ml empty syringe, thus lowering the negative pressure inside SE to a ~10% or less of the "suction" level. The lengths of CC ends sucked into the SEs did not exceed 1–1.5 mm (S1 Fig). Care was taken not to stretch the CCs during the procedure. A single CC was recorded at a time, and all CCs were positioned in SEs symmetrically, with their medial part in the middle.

The SEs used for "whole" CC recording and stimulation (Fig 1C and S1 Fig) were similar to those used by others for optic nerves [36,38,40,44], and were prepared from borosilicate glass (1.5 mm o.d., 1.0 mm i.d.), with tips fire-constricted to 0.3 mm i.d. to match the size of "whole" CC ends.

The stimulating pulses of 0.1 ms duration and varying amplitude were applied via the PSIU6 stimulus isolation unit of Grass S88 dual-channel stimulator (Grass Technologies). CAPs were recorded in DC mode with Axoprobe 1A amplifier (Axon Instruments / Molecular devices, USA). The signals, along with stimulation pulses recorded from the “Monitor” output of the stimulator, were processed using pClamp8 software and DigiData 1320A at a 100 kHz low-pass filter (all from Axon Instruments / Molecular Devices, USA). Data analysis was performed offline using Clampfit 10 Data Analysis Module of pClamp10 software after filtering the signals to 10 kHz low-pass range. Digital averaging of maximal CAPs from sets of experiments was performed using the Analyse / Concatenate Files function of Clampfit 10, followed by Analyse / Average Traces function of the same program.

The electrical resistances of recording electrodes were measured by applying 1 ms * 1 nA current pulses through the Axoprobe 1A amplifier, triggered by Clampex 10 and measured by the recorded voltage response, with the bridge balance of the amplifier set to 0. The resistances of SE electrodes, filled with aCSF (see Solutions) and measured with CC inside, were 0.07 ± 0.004 MΩ, which was significantly lower compared to 0.65 ± 0.1388 MΩ of MEs that had tip diameters 2–3 μm and were filled with 3M NaCl.

The electrical noise of SE and ME recordings was compared using peak-to-peak and RMS (root mean square) values measured in the baseline of recorded CAPs. The peak-to-peak values were measured using the Statistics function of Clampfit 10, and the RMS noise was measured using the Power Spectrum / Barlett function of Clampfit 10. Both parameters showed nearly twice lower noise of SE recordings compared to ME recordings, which were (mean ± standard error of mean, SEM) 0.034 ± 0.0003 mV for SE as compared to 0.073 ± 0.0102 mV for ME for peak-to-peak noise and 0.006 ± 0.0002 mV for SE as compared to 0.012 ± 0.0016 mV for ME for the RMS noise (see also S2 and S3 Figs).

### Solutions

The sucrose-substituted dissection aCSF contained (in mM): 210 sucrose, 26 NaHCO_3_, 2.5 KCl, 1 CaCl_2_, 4 MgCl_2_, 1.25 NaH_2_PO_4_, and 10 D-glucose (315 mOsmol), pH 7.4 after equilibration with 95%O_2_+5%CO_2_ (carbogen).

The standard aCSF used for slice or "whole" CC incubation and recording contained (in mM): 125 NaCl, 2.5 KCl, 1.25 NaH_2_PO_4_, 2 CaCl_2_, 1.3 MgSO_4_, 26 NaHCO_3_, and 10 D-glucose (308 mOsmol), pH 7.4 after saturation with carbogen.

Potassium channel blocker 4-aminopyridine (4-AP) was added to standard ACSF on the day of experiment from stock solutions kept at 4°C. For modeling ischemia by OGD, glucose was equimolarly replaced with sucrose, and the solution was equilibrated with 95%N_2_+5%CO_2_ instead of carbogen.

All chemicals were obtained from Sigma-Aldrich Canada (Oakville, Ontario).

### Statistics

Statistical comparisons between two groups were done using SigmaStat 3.5 (Systat Software, Inc) using t-tests with Mann-Whitney Rank Sum Test in cases when normality test failed. Values in text are shown as mean ± s.e.m. (standard error of mean); error bars in plots represent s.e.m.. P values <0.01 and <0.001 are shown in graphs as ** and *** respectively; for p values >0.05, numerical values are shown.

## Results

### General description of compound action potentials of "whole” CC

As shown in Fig 1B and 1C, both the SE-recorded "whole" CC and ME-recorded CAPs exhibited two clearly separated peaks (N1 and N2 in ME, recordings, Fig 1B; peak 1 and peak 2 in SE recordings, Fig 1C), which represent the activity of myelinated and non-myelinated axons, respectively. The positive polarity of "whole" CC CAP peaks is in accordance with CAPs recorded with SEs from peripheral nerves or the optic nerve [38–40,45–48].

SE recording of "whole" CC CAPs has several advantages over ME recordings, producing CAPs with larger amplitudes at comparable conduction distances and better separation from stimulus artifacts. The signal-to-noise ratio of maximal CAPs showed advantages of our approach, with mean values of 32.5 ± 1.48 for SE compared to 14.7 ± 1.33 for ME for peak 1, and 4.8 ± 0.62 for SE compared to 2.9 ± 0.36 for ME for peak 2 (see S2 and S3 Figs).

In ME recordings, the relative sizes of peaks N1 and N2 vary broadly depending on positioning of the ME tip, while SE-recorded "whole" CC CAPs are devoid of this bias. The peak amplitudes of maximal ME- and SE-recorded CAP peaks at 1.5–2 mm conduction distances are compared graphically in Fig 1D, which also shows the range of recorded CAP peak amplitudes. The mean amplitude (mean ± s.e.m.) of SE-recorded peak 1 was 2.71 ± 0.54 mV (n = 11), statistically different (p = 0.003) from 1.13 ± 0.10 mV (n = 13) amplitude of N1 peak of ME-recorded CAPs. For SE-recorded peak 2 and ME-recorded N2 peak, the amplitudes were 0.64 ± 0.14 mV and 0.30 ± 0.04 mV, respectively (p = 0.010). The peak2/peak1 amplitude ratio in SE-recorded CAPs was 0.23, and the N2/N1 amplitude ratio of ME-recorded CAPs was 0.27.

**Fig 2:**
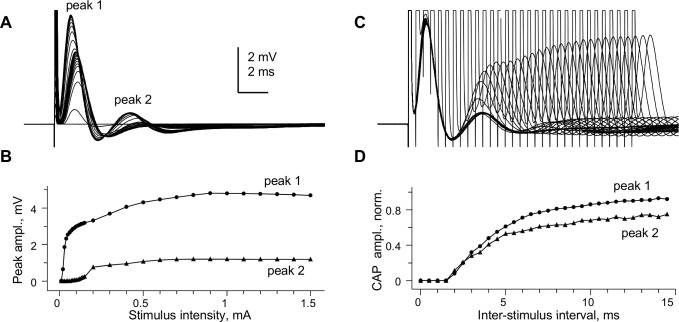
Stimulus-response relationship and refractoriness of “whole” CC compound action potential peaks. This figure shows further details on “whole” CC CAP from in Fig 1C. (A) Superimposed CAPs recorded at varying stimulus intensities. (B) stimulus-response plot of amplitudes of peaks 1 and 2 for CAPs shown in (A). (C) Superimposed CAPs evoked by paired stimuli of varying intervals, with 0.5 ms interval increments. Individual traces in (B) and (C) are averaged from 6 consecutive recordings at each stimulation intensity, taken at 10 s intervals. (D) Plot of peak amplitudes of 2^nd^ CAP in the pair as a function of inter-stimulus interval, normalized to peak amplitudes of the 1^st^ CAP in the pair. The absolute refractory period of both peaks is 1.5 ms, while the relative refractory period of both peaks exceeded 15 ms. Conduction distance 1.5 mm; room temperature.

The SE-recorded "whole" CC CAPs could be finely graded by stimulation intensity (Fig 2A), and the slower-conducting peak 2 had a distinctly higher threshold, as shown in Fig 2B. Fig 2C illustrates the refractoriness of "whole" CC CAP peaks, tested by paired maximal stimuli of varying intervals. The amplitudes of peaks 1 and 2 of the 2^nd^ CAP in the pair, measured after subtracting the underlying 1^st^ CAP, are shown plotted vs. inter-stimulus interval in Fig 2D, showing similar absolute refractory periods of 1.5 ms.

SE recording of "whole" CC CAPs has clear advantages over ME recording at longer conduction distances. Our attempts to record CAPs with MEs at 3–4 mm or longer distances produced signals in the low microvolt range that, although could be further resolved by signal averaging, did not deem practical due to thinning of the CCs with distance. The important advantages of SE CAP recording from CC at distances longer than 2 mm are the larger amplitudes of CAPs and the better separation of peaks 1 and 2.

The rest of experiments described in this paper were entirely focused on our novel “whole” CC CAP recordings.

### Rostro-caudal differences of "whole" CC compound action potentials

The amplitude of CAPs, which reflects the number of contributing axons, depended on the distance between stimulating and recording electrodes. In coronal brain slices, the number of axons running for sufficient distances within the plane of a slice (and hence within its corresponding "whole" CC) varies depending on the rostro-caudal position and the distance between stimulation and recording sites, affecting the size of recorded CAPs.

**Fig 3:**
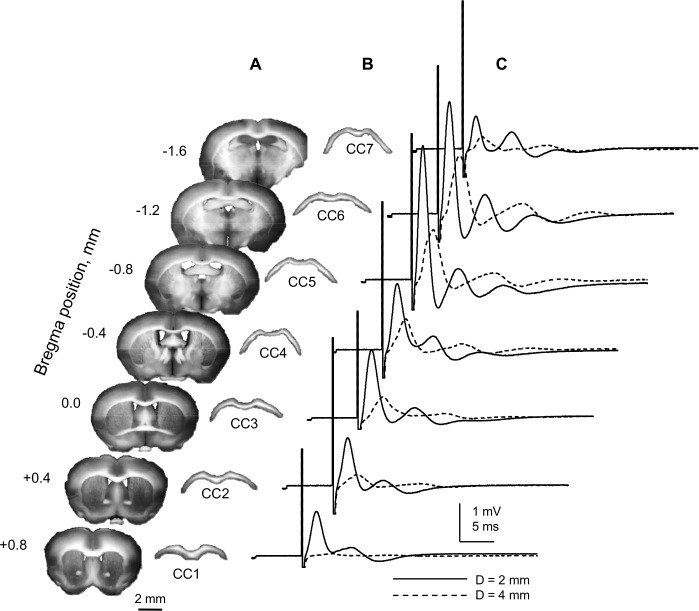
Rostro-caudal patterns of “whole” CC CAPs compound action potentials. (A) Seven consecutive 400 μm-thick coronal slices from a single mouse brain. (B) CCs isolated from slices shown on (A). The rostral-most coronal slice was the first from rostral end of the brain where the CC had a continuous appearance across the midline, and its corresponding isolated CC is referred to hereafter as CC1. The rostro-caudal position of the slice from which CC3 was isolated corresponds approximately to bregma 0, identified by the presence of anterior commissure in the lower part of the slice, continuously running across the midline. (C) Suction electrode (SE) recordings from CCs shown in (B). Maximal CAPs from each CC, recorded at 4 mm (dashed traces) and 2 mm (solid traces) conduction distance. The recordings started at 4 mm, followed by drawing the CCs deeper into the SE’s to reach a 2 mm conduction distance, while keeping the arrangement symmetrical around the CC mid-point. The smaller amplitude of CAPs recorded at 4 mm distance likely reflects a lower number of axons in continuity between the stimulated and recording sites, in agreement with decreased CAP areas. Each trace represents an average of 6 consecutive recordings taken at 10 s intervals. All recordings shown here were done at room temperature.

Fig 3 shows a sequence of 7 coronal brain slices, their corresponding “whole” CCs and CAPs of "whole" CCs recorded with SE in each slice at two conduction distances, 2 mm and 4 mm (solid and dashed traces, respectively). The CAP amplitudes varied between slices in rostro-caudal direction in a standard pattern (Fig 3C). Increasing the distance between stimulating and recording sites resulted in a sharp decline of recorded signals, reflecting the reduced number of axons in continuity between the stimulation and recording sites. As illustrated in Fig 3C, the CAP amplitudes were largest at CC5-CC6 (bregma -0.8…-1.2), and were larger at 2 mm (2.5–3 mV for peak 1) compared to 4 mm distance (< 1.5 mV for peak 1) between stimulation and recording sites.

Because longer conduction distances allow for better separation of CAP components by their arrival times (hence, conduction velocities; CV) in this paper we focused on "whole" CC CAPs recorded at 4 mm conduction distance, with symmetrically positioned stimulating and recording SE’s.

**Fig 4:**
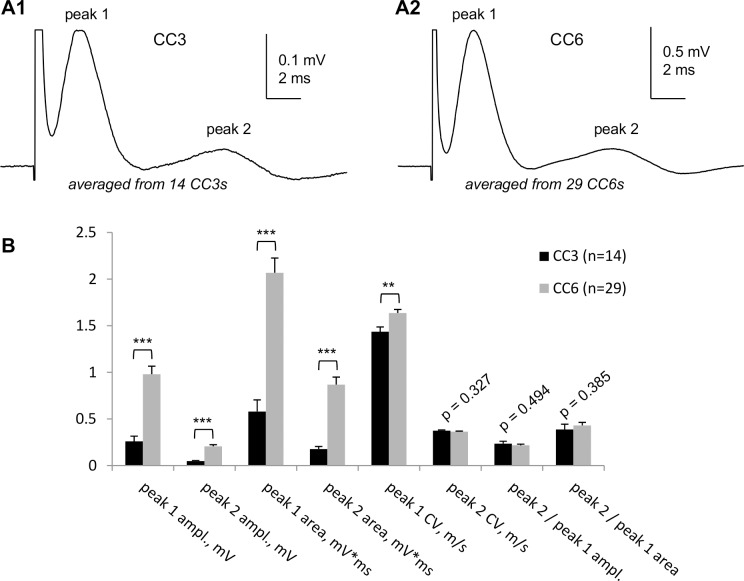
Detailed characteristics of “whole CC CAPs recorded at 4 mm conduction distance from CC3 and CC6. (A1, A2) Digitally averaged “whole” CC CAPs recorded from 14 CC3s (A1) and 29 CC6s (A2) from different mouse brains. The CC3 and CC6 were identified as explained in Fig 3. Note different voltage scales in A1 and A2. (B) Detailed statistical comparison of “whole” CC3 and CC6 CAP parameters: ampl.—amplitude; CV—conduction velocity; peak2/peak1 ampl.—ratio of peak amplitudes; peak2/peak1 area—ratio of peak areas. ** p<0.01; *** p<0.001. All recordings were performed at room temperature.

Fig 4 compares in detail the “whole” CC CAPs recorded from CC3 and CC6 at 4 mm conduction distance. The longer-latency peak 2 was invariably smaller than peak 1, comprising 0.24 ± 0.03 of peak 1 amplitude in CC3 (n = 14) and 0.22 ± 0.01 of peak 1 amplitude in CC6 (n = 29). The amplitude or area peak2/peak1 ratio did not statistically differ between CC3 and CC6 (Fig 4B). Note that both the amplitude and area of peak 2 were around 0.2–03 of peak 1, as invariably observed also in individual recordings shown in other figures of this paper.

The CV of CAP peaks was calculated by dividing the conduction distance by peak latency. At room temperature where most measurements were done, the CV of peak 1 was 1.64 ± 0.04 m/s in CC6 (n = 29) and 1.43 ± 0.05 m/s in CC3 (n = 14); difference statistically significant (p = 0.004), suggesting differences in structural features of myelinated axons. For peak 2, the CV was 0.36 ± 0.01 m/s in CC6 (n = 29) and 0.37 ± 0.03 m/s in CC3 (n = 14), difference statistically insignificant (p = 0.327). With the Q_15_ temperature coefficients of 1.99 for peak 1 and 2.06 for peak 2, determined later in this paper, the CVs of peak 1 and peak 2 at physiological temperatures are projected to 2.8 m/s and 3.3 m/s for peak 1 (CC3 and CC6, respectively) and to 0.74–0.76 m/s in both CC3 and CC6 for peak 2.

### Validating the "myelinated" nature of peak 1 of "whole" CC CAPs using dysmyelinated *shiverer* mice

**Fig 5:**
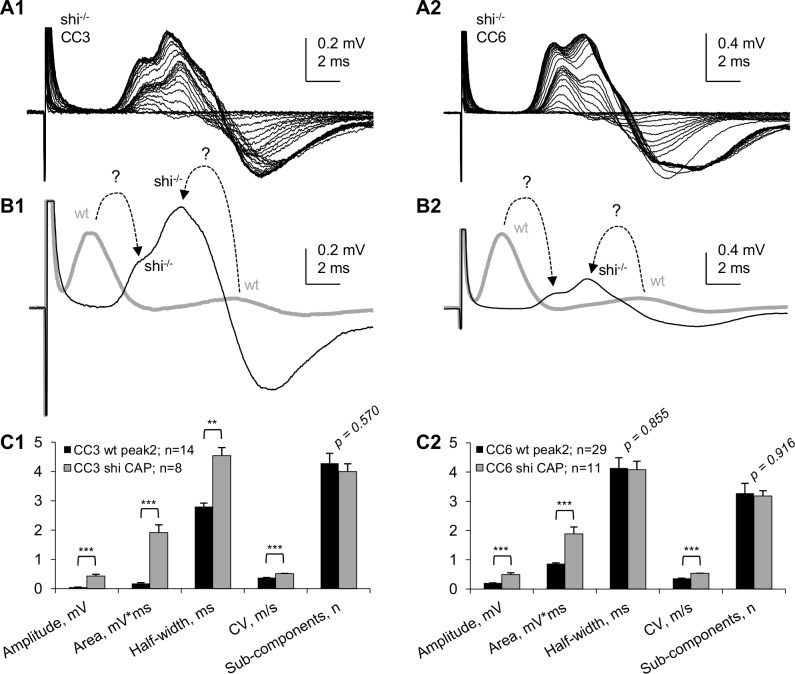
Comparison of “whole” CC CAPs of wild type (wt) and dysmyelinated *shiverer* (shi^-/-^) mice. (A1, A2) Superimposed “whole” CC CAPs recorded at varying stimulation intensities in CC3 (A1) and CC6 (A2). Each trace in A1 and A2 represents an average of 6 consecutive recordings taken at 10 s intervals. (B1, B2) Digitally averaged “whole” CC CAPs recorded from wt (thick gray traces) and shi^-/-^ (thin black traces) CC3 (B1, digitally averaged from 8 experiments) and CC6 (B2, digitally averaged from 11 experiments). CAPs from wt CCs, (digitally averaged, from Fig 4A1 and 4A2; brought to same scale with shi^-/-^ CAPs) are shown in B1 and B2 as thick gray traces. Dashed arrows with question marks show hypothetical relationships between peaks of wt and shi^-/-^ CAPs (see text). (C1, C2) Statistical comparison of main parameters of wt and shi^-/-^ “whole” CC CAPs. ** p<0.01; *** p<0.001.

To validate the peak 1 of “whole” CC CAPs as reflecting myelinated axons, we conducted similar SE-recording experiments on "whole" CCs from *shiverer* mice (shi^-/-^), a myelin-deficient mutant used extensively in other studies from our group [26,49–51]. Examples of “whole” CC CAPs of shi^-/-^ mice and their comparison with wt are shown in Fig 5. The shi^-/-^ “whole” CC CAPs totally lacked the fast peak 1 characteristic of wt in both CC3 and CC6, however they exhibited a slower complex CAP that had two distinct components (Fig 5A1 and 5A2). Fig 5B1 and 5B2 shows superimposed digitally averaged “whole” CC CAPs from wt and shi^-/-^ mice, with dashed arrows showing hypothetical relations between the peaks of wt and shi^-/-^ CAPs. The absence of a fast peak 1 in shi^-/-^ “whole” CC CAPs confirms that in wt it is generated by myelinated axons. The pronounced negative phase of shi^-/-^ CAPs may be due to slower signal propagation within the SEs, similar to biphasic CAPs observed at premyelinating stages of development in the optic nerve (see Discussion). In our SE recordings from wild type CCs, the biphasic shape was typical for the slower conducting peak 2 compared to peak 1, as seen in other figures of this paper. While this biphasic shape may result in some degree of distortion of true peak latencies, the presence of two positive peaks in shi^-/-^ CAPs evoked by varying stimulation intensities (Fig 5A1 and 5A2) clearly indicates two major axonal populations in *shiverer* mice, dysmyelinated and non-myelinated, differing in their stimulation thresholds and conduction velocities.

An unexpected finding in this study was that peak 2 also changed in shi^-/-^ mice where it had shorter latency and higher amplitude compared to wt. Because dysmyelination is unlikely to directly affect the non-myelinated axons, a possible explanation for this change could be compensatory changes in non-myelinated axons of shi^-/-^ mice. Because it is not clear if the faster peak of shi^-/-^ CAPs represents dysmyelinated axons or a sub-population of non-myelinated axons with more pronounced compensatory changes, we only compared the overall characteristics of shi^-/-^ CAPs with those of peak 2 of wt CAPs, summarized in Fig 5C1 and 5C2.

### Temperature dependence of compound action potentials of "whole" CC

SE-recording of "whole" CC CAPs at room temperature (20–22°C) is suitable for better separation of CAP peaks due to slowing down their CVs, further facilitated by longer (4 mm) conduction distance.

**Fig 6:**
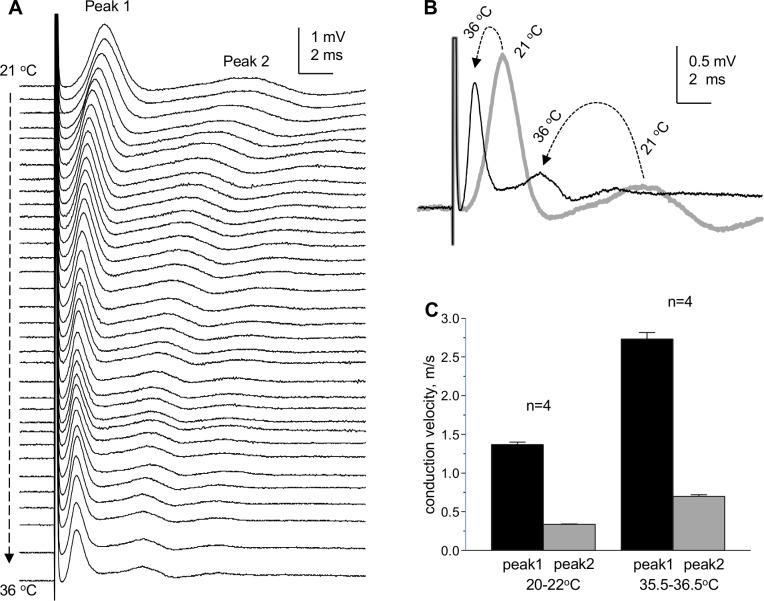
Temperature dependence of “whole” CC CAPs. (A) Superimposed “whole” CC CAPs recorded during the increase of the temperature of perfusing solution from 21°C to 36°C. (B) Superimposed CAPs recorded at 21°C (thick gray trace) and 36°C (thin black trace). The dashed arrows show the changes of CAP peaks from room to high temperature. The CAPs shown in this figure were recorded without averaging to reflect the changes during the temperature test. From these experiments, we estimated the Q_10_ of conduction velocities of peak 1 (myelinated axons) and peak 2 (non-myelinated axons) as 1.33 and 1.37, respectively.

In a separate set of experiments, we tested the effects of increasing the bath temperature from room to physiological levels. Increasing the bath temperature resulted in sharp reduction of CAP peak latencies and widths (Fig 6), indicating increased CVs and higher synchrony of arriving action potentials at recording site. The hyperpolarizing shift of CAP baseline (the "compound resting membrane potential" or CRMP) seen in Fig 6A was reversible upon return to room temperature (not shown). The temperature-dependent changes of CRMP suggest that at room temperature the membrane potentials of axons are depolarized compared to physiological temperatures, as should be expected due to reduced activity of electrogenic membrane pumps.

To project the measured CVs to physiological range, in selected experiments we determined the temperature coefficients (Q_10_'s and, for practical considerations, Q15’s) of CVs in myelinated and non-myelinated axons. The temperature-dependent changes of CVs of CAP peaks 1 and 2 were studied in 8 individual “whole” CCs and are summarized in Fig 6C. The CV of peak 1 was 1.37 ± 0.03 m/s at room temperature (range 1.23–1.48 m/s) and 2.73 ± 0.08 m/s at physiological temperature (range 2.39–3.09 m/s). For peak 2, the CV was 0.34 ± 0.01 m/s at room temperature (range 0.32–0.37 m/s) and 0.70 ± 0.02 m/s at physiological temperature (range 0.63–0.81 m/s). From these measurements, the Q_10_ of CV of myelinated axons was estimated to be 1.33, which is close to 1.4 values found for myelinated axons in rat optic nerve [52] and 1.46 values can be calculated from CAP CVs measured in rat spinal cord dorsal white matter at 25°C and 37°C [53]. Similarly, we estimated the Q_10_ of CV of non-myelinated axons in CC to be 1.37, which is close to 1.6 value estimated in hippocampal Schaffer collaterals [54]. For practical considerations, we also estimated Q_15_ values for recalculation of CVs from room to physiological temperature: 1.99 for peak 1 and 2.06 for peak 2.

The reduced amplitudes of CAPs at physiological temperatures were a common phenomenon and were reversible upon return to room temperatures, along with return of CAP baselines to more depolarized levels.

### Effects of modelled ischemia on compound action potentials of “whole” CC

**Fig 7:**
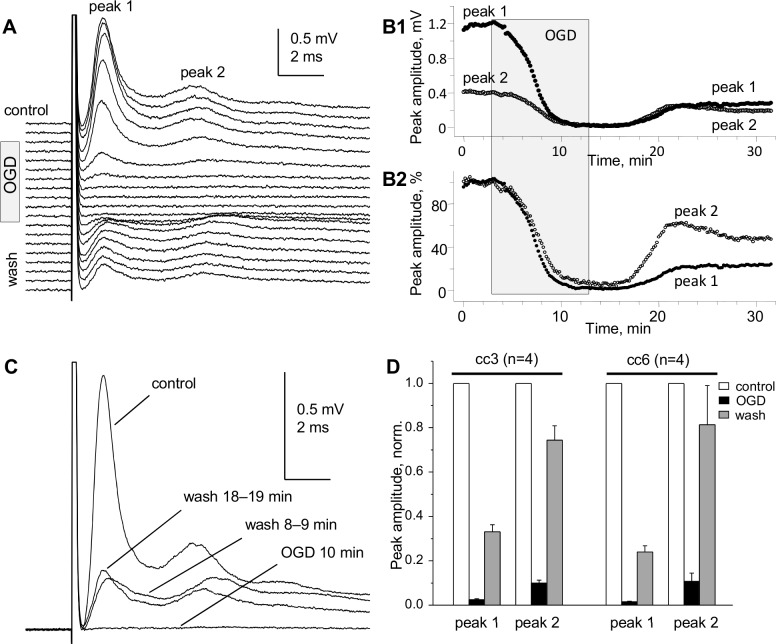
Pronounced effects of oxygen glucose deprivation (OGD) on “whole” CC CAPs. (A) Superimposed CC CAPs recorded before (control), during OGD and during washout. (B1, B2) Time course of changes of absolute (B1) and normalized (B2) amplitudes of peaks 1 and 2 during 10-minute OGD and 20-minute washout. (C) Superimposed CAPs before, during and after OGD. (A-C) Representative data from a single experiment on CC6. The CAPs were recorded without averaging to reflect the fast changes during the test. (D) Summary of OGD–induced changes of CAP peaks after 10 min OGD and 20 min washout in CC3 and CC6. Temperature 35.5°C–36.5°C.

The isolated CC provides better conditions for diffusional exchange with bath media compared to CC in brain slices. Here we tested the effects of ischemia modeled by OGD (see Solutions in Methods). The OGD, studied at 35.5–36.5°C bath temperature, had pronounced and fast effects on SE-recorded “whole” CC CAPs, resulting in sharp decrease of CAPs to near complete disappearance within 7–8 minutes of OGD. The half-decay time (decay to 50% of control CAP peak amplitudes) of “whole” CC CAP peak amplitudes after the onset of OGD was 2–3 minutes, as illustrated in Fig 7. The recovery of CAPs upon washout from 10-minute OGD was incomplete within 20–40 minutes of observation, and showed differential time course of recovery of myelinated (peak 1) and non-myelinated (peak 2) axons, with peak 2 recovering faster and to larger extent compared to peak 1 (Fig 7A–7C). A summary of eight OGD experiments (four on CC3 and four on CC6) is shown in Fig 7D, further illustrating the higher sensitivity of myelinated compared to non-myelinated axons to 10-minute OGD, i.e. significantly shorter exposure to OGD compared to ME CC studies using brain slices [27,28,55,56].

### The complexity of "whole” CC CAPs revealed by potassium channel blocker 4-AP

The classical *in vivo* ME study in rats by Preston, Waxman & Kocsis [10] demonstrated a marked increase of the amplitude and duration of the second (non-myelinated) peak of CAP after local injection of 4-AP, which contrasted the little or no sensitivity of the fast (myelinated) peak. Here, using SE “whole” CC recording, we confirm this differential effect of 4-AP on myelinated and non-myelinated axons.

**Fig 8:**
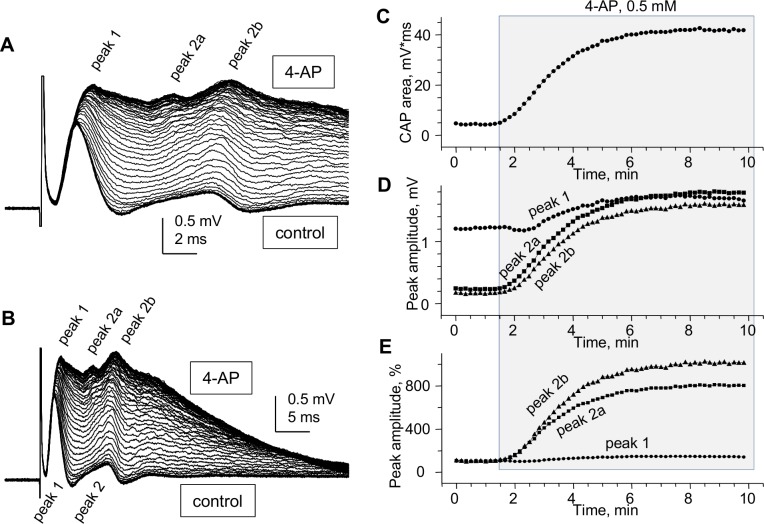
Pronounced effects of potassium channel blocker 4-aminopyridine (4-AP) on “whole” CC CAPs. (A and B) Superimposed “whole” CC CAPs recorded from CC6 during administration of 0.5 mM 4–AP, shown at two different time scales to illustrate the pronounced prolongation of CAP decay. Note the two visually identifiable slow peaks (peak 2a and peak 2b) at the maximal effect of 4-AP, emerging by gradual changes of peak 2 shape from control to 4-AP. The CAPs were recorded without averaging to reflect the fast changes during the test. (C-E) Time course of changes of CAP area (C) and peak amplitude (D, absolute; E, normalized) during administration of 4-AP. The half-maximum of the effect of 4-AP was reached within 3.5–4 minutes. The effects were reversible (not shown). Room temperature.

As seen in Fig 8A and 8B, 4-AP not only caused an overall sharp increase of CAP amplitude and duration, but also helped accentuate distinct peaks that were barely distinguishable before the drug administration. Most noticeably, 4-AP caused an increase in amplitude and prolongation of peak 1 (Fig 8A) and appearance of distinct slower conducting peaks of the non-myelinated group, marked as peak 2a and peak 2b in Fig 8A, and even slower peaks. The time course of overall CAP area and of peaks 1, 2a and 2b for this experiment is shown in Fig 8C–8E. The pronounced effects of 4-AP may be due to better penetration of the drug into isolated CCs compared to studies in brain slices, and may reflect repetitive firing of axons or the possible facilitation of hidden CAP peaks.

### A third peak of compound action potential of “whole” CC

**Fig 9:**
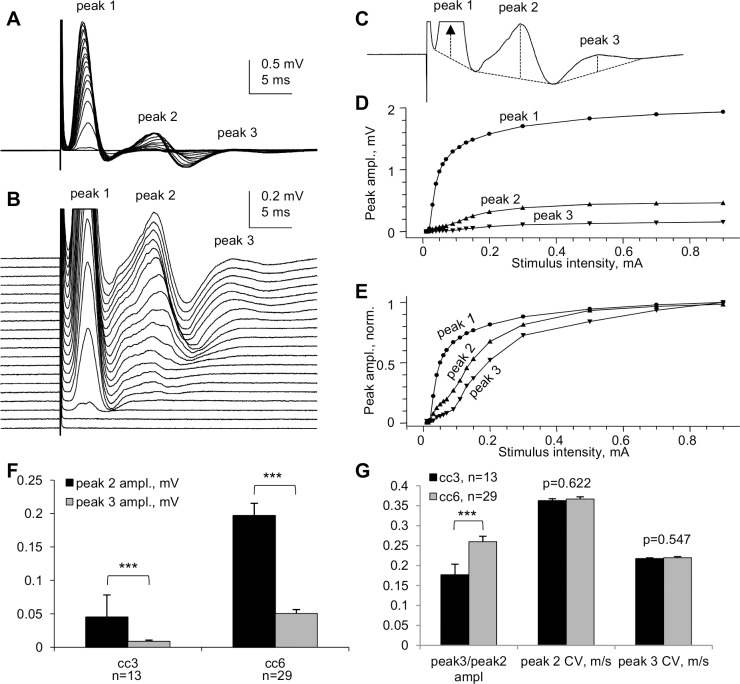
A third peak of “whole” CC CAPs. (A) Superimposed CAPs evoked by varying stimulation intensities in an experiment on CC6. (B) Same traces as in (A), shown at higher gain and separated vertically to illustrate the pattern of appearance of peak 3 at higher stimulation intensities. Each trace in A and B represents an average of 6 consecutive recordings taken at 10 s intervals. (C) Top (maximal) CAP from (B); dashed lines explain the measurement of the amplitudes of peaks 1, 2 and 3 from their projected bases. (D–E) Stimulus–response plots of the amplitudes of peaks 1, 2 and 3 shown in absolute (D) and normalized (E) values to illustrate different thresholds of CAP peaks, and specifically the higher threshold of peak 3 compared to peak 2. (F) Statistical comparison of the amplitudes of CAP peaks 2 and 3 in CC3 and CC6. (G) Comparison of peak3/peak2 amplitude ratios (peak3/peak2 ampl) and conduction velocities (CVs) of peaks 2 and 3 in CC3 and CC6. Room temperature. *** p<0.001.

Besides the major peaks 1 and 2, "whole" CC CAPs exhibited a slower, smaller peak 3 that was more pronounced in more caudal CCs from the CC1…CC7 series (Fig 3C). Fig 9 illustrates the peak 3 in a CC6 slice and provides statistical comparisons of peaks 2 and 3 in CC3 and CC6.

A profound negative 3rd peak of ME-recorded CC CAPs had been observed in an *in vivo* study in rats [10] where it was proposed that it could be relayed through cortical synapses and then redirected through the CC toward the contralateral hemisphere. Because in the present study the CCs were completely isolated from gray matter, it is unlikely that the 3^rd^ peak could be a reverberating signal. We therefore conclude that the 3^rd^ peak of "whole" CC CAPs, as well as the third negative peak observed by others in ME recordings in brain slices, represents a distinct population of smaller caliber non-myelinated CC axons, which may be prevalent in caudal parts of the brain.

## Discussion

This paper describes a novel approach for electrophysiological studies of myelinated and non-myelinated axons of CNS white matter of laboratory animals in normal and pathophysiological conditions. The isolation of corpus callosum from brain slices provides better diffusional exchange with perfusing media compared to brain slices, allowing for efficient drug delivery or modeling short-term ischemia as demonstrated here with K channel blocker 4-aminopyridine and 10-minute oxygen-glucose deprivation. Recording compound action potentials (CAPs) from isolated corpus callosum with our approach using suction electrodes allows for more representative data due to recording from larger axonal populations and provides the following major advantages:

1. Unlike microelectrode recordings where the relative sizes of CAP peaks reflecting myelinated and non-myelinated axons (respectively, peaks 1 and 2) vary broadly depending on electrode position, our approach provides stable and highly reproducible ratios of the sizes of peaks 1 and 2. This provides a quantitative approach for electrophysiological estimates of relative sizes of myelinated and non-myelinated axonal populations of corpus callosum in different normal and pathological experimental conditions including demyelination/remyelination, developmental aspects and aging of the CNS white matter. While this parameter may depend on possible changes of action potentials in individual axons, its low variability compared to microelectrode studies may be useful for characterizing the two axonal populations in distinct physiological or pathological conditions.
2. An improved and multi-parameter analysis of CC CAPs due to (a) lower noise and higher signal-to-noise parameters, (b) better separation of peak 1 from stimulus artifact, (c) less complicated shape of CAPs: mostly biphasic peaks (and almost monophasic peak 1), as compared to profoundly triphasic overlapping peaks 1 and 2 recorded by microelectrodes and (d) ability to record at longer conduction distances, with better separation of the peaks.
3. A highly sensitive comparison of CAPs along the rostro-caudal axis (see Fig 3) and determination of conduction velocities of CAP peaks, revealing subtle differences in conduction velocities of myelinated (but not non-myelinated) axons between different rostro-caudal positions, such as the illustrated two rostro-caudal positions separated by as little as 1.2 mm shown in Fig 4. Uncovering such differences with microelectrode recordings would be a very time- and labor-consuming task.
4. Unlike many papers showing apparently arbitrarily selected CAP traces to illustrate specific points, we introduce digitally averaged CAPs (Fig 4A1 and 4A2; Fig 5B1 and 5B2), that, along with recording from larger populations of axons, are devoid of such bias.
5. For the first time, we show that in dysmyelinated shiverer mice, the CAPs are not reduced to a merely single-peak shape, as was thought earlier based on microelectrode recordings [26], but in fact exhibit a complex shape with two major peaks (Fig 5) that apparently reflect two major axonal populations: dysmyelinated and non-myelinated. We present this novel finding to illustrate the higher sensitivity of our approach compared to microelectrode recording in slices, and to validate the peak 1 as “myelinated” (detailed further in item (8) below). These experiments show that our approach may have broad implications for studies of various pathological conditions involving demyelination, and their treatments.
6. We have demonstrated the presence of a third peak in CAPs (Fig 9) that was seen in some published illustrations of microelectrode recordings but not discussed, except for the classical *in vivo* study of Preston et al 1983 [10] who suggested that this peak could represent a reverberating of signal via the cortex. Our finding of peak 3 in completely isolated corpus callosum rules out the reverberation and suggests the presence of a much slower conducting non-myelinated axonal population.
7. Comparative determination of temperature coefficients (Q_10_, Q_15_) of conduction velocities of myelinated and non-myelinated axons. Earlier reports have done such assessments in the CNS on only one of these axon types, in different studies on different structures: the optic nerve (myelinated) [52] and the hippocampal Shaffer collaterals (non-myelinated) [54]. We took advantage of both types of axons being present in the same preparation, and estimated their temperature dependence simultaneously within the same tissue samples and in same experimental conditions. From these experiments, we estimated the Q_10_ of conduction velocities of peak 1 (myelinated axons) and peak 2 (non-myelinated axons) as 1.33 and 1.37, respectively. For practical reasons, Q_15_ values of 1.99 for peak 1 and 2.06 for peak 2 to project room temperature measurements to physiological temperatures and vice versa.
8. Validation of peak 1 of suction electrode-recorded CAPs as myelinated (see also item (5) above). Because we introduce a novel way of recording from corpus callosum, we used dysmyelinated shiverer mice and showed that this peak disappears from its usual position in the CAP, and is replaced with a longer latency peak, apparently reflecting the slower conducting dysmyelinated axons. We further found unexpected changes in the latency of peak 2 (non-myelinated axons), which surprisingly became shorter in shiverer mice, suggesting compensatory changes in conduction properties of non-myelinated axons, never thought of before.

While the present study is focused on the corpus callosum (CC), a similar approach using improved resolution suction electrode recordings from dissected white matter strips is applicable to other CNS white matter structures. This may be particularly important in the light of recent advances in MR tractography and connectomics [1–7] and accumulating knowledge on the structural framework of brain wiring, that now require adequate data on functional properties of axons in white matter pathways. Using "net white matter" preparations such as the isolated CCs described in this paper allows for detailed electrophysiological characterization of axonal conduction without complications by reverberating signals from other brain structures. Advancing the knowledge on functional properties of axons in cerebral white matter structures is important for diagnosis and treatment of a variety of brain disorders including stroke, traumatic brain injury, demyelinating, neurodegenerative and other conditions.

Our approach using SE recording of compound action potentials (CAPs) with improved separation of components reflecting myelinated and non-myelinated axons in "nerve-like" white matter strips isolated from brain slices provide a suitable alternative to laborious microelectrode (ME) recordings, allowing for detailed characterization of distinct axonal populations.

Using isolated white matter strips has all advantages of *in vitro* slice recordings by controllable changes of the extracellular media such as administration and washout of pharmacological drugs, modeling ischemia and studying temperature dependence, illustrated in this paper. Furthermore, this approach is particularly useful for studying axonal function in demyelination models, as illustrated by recordings from dysmyelinated *shiverer* mice CCs, revealing features not detected in earlier ME-recording studies, as discussed in detail below.

Our novel approach raises the need for critical evaluations of its advantages and limitations in comparison with existing approaches used for assessments of the functional properties of myelinated and non-myelinated axonal populations of corpus callosum, as discussed in separate sections below.

### The novelty and advantages of "nerve-like" CC preparation

Unlike studies with field ME recording within CC [10,11,13–17,32,33,57–62] or from cortical neurons [22,63–68] in response to stimulation of CC [63–68] or contralateral cortex [64–67], our approach brings the electrophysiological studying of CC axonal populations to "nerve-like" experimental conditions. In the CNS, "nerve-like" electrophysiological studies have been extensively used on the optic nerve [34–40,44,47,69] and on spinal cord white matter by others [47,70–77] and by our group [51,78–83] but never applied to other white matter structures.

ME-recorded CAPs have complex shapes due to partial overlap of two triphasic peaks corresponding to action potentials of myelinated and non-myelinated axons propagating through the recording site. SE-recorded CAPs, with biphasic rather than triphasic shapes of their peaks (and the far less biphasic peak 1 compared to ME recordings) provide important advantages for electrophysiological identification and analysis.

Quantitative descriptions of ME-recorded CAPs typically focus on negative peaks N1 and N2 for assessing axonal conduction in the CC. Due to typically short conduction distances in ME recordings within CC (1–2 mm; but see 3.3 mm distance in [10]), the faster N1 peak if often markedly distorted by the overlapping tail of stimulus artifact, forcing the researchers to use room temperatures to slow down the axonal conduction for a better separation from stimulus artifact. A sharp decline in the amplitude of ME-recorded CC CAPs with increasing the distance between stimulation and recording sites from 0.5 mm to 2.5 mm in mouse brain slices (see e.g. Fig 3 in [15]) is a major limiting factor for using MEs for longer distance recordings.

Suction electrode (SE) recordings from "whole" CC allow for better separation of CAPs from stimulus artifacts, with far less distortion of the fast component compared to ME recordings. At distances of 1.5–2 mm, commonly used for ME CC CAP recording, the amplitudes of "whole" CC SE-recorded CAPs have more than two-fold amplitudes compared to ME recordings. The amplitude of SE-recorded CAPs depends on the electrical resistance of SE with the nerve inserted, as discussed in [40]. We monitored this resistance by measuring the voltage responses of SE with nerve using 1 nA x 1 ms current pulses in every experiment, and did not find large variations that could account for CAP amplitude variability. Despite this variability, the stimulation of "whole" CCs with SEs as opposed to surface electrodes used in ME recording, combined with "whole" CC SE recording provides a more adequate covering of the whole axonal population compared to ME recordings. An important advantage of SE "whole" CC over ME slice or *in vivo* recording is that ME recording highly depends on fine positioning of recording electrode and its alignment with stimulated axonal bundles, which is not required in SE recording.

An important advantage of "whole" CCs for electrophysiology is the improved separation and more consistent relative amplitudes of CAP components reflecting the myelinated and non-myelinated axonal populations. By allowing CAP recordings at longer distances, the "whole" CCs are particularly useful for a better separation of CAP peaks reflecting the conduction in myelinated and non-myelinated axonal populations.

### Limitations of "whole" CC CAP recording

As in any SE stimulation and recording, the successful use of "whole" CCs depends on the precise matching of the tissue drawn into SE's with the SE inner diameters [40], and on other variables such as the number and caliber of axons running in continuity between the stimulated and recorded sites. A common limitation of SE recording is the lack of true monophasic shapes of recorded CAPs due to propagation of action potentials within the SE portion of axons, resulting in different degrees of biphasicity depending on preservation of conduction properties of distinct types of axons within the SE. As in any SE-recorded CAPs, the signals propagating within the suction electrode portion of axons may create a time-shifted signal of opposite polarity affecting the peaks and valleys of recorded signals, being more pronounced in slower conducting components (seen to different extent in Figs 2–9). This is in agreement with the pronounced biphasic shapes of SE-recorded CAP peaks of slower conducting C-fibers of peripheral nerves [84,85] and premyelinating and more homogenous axonal populations of optic nerves in early postnatal development [36,86,87]. However, these common limitations of SE recording do not negate the overall advantages of SE recording of CC CAPs shown in this paper, which demonstrates much simpler shapes of the signals compared to complex overlapping time-shifted triphasic components of ME-recorded CC CAPs.

Unlike nerves where axons run for long distances without deviating from the nerve trunk, the deviation of axons between stimulated and recorded sites in "whole" CCs obtained from flat coronal slices as described in this paper constrains certain limitations on successful CAP recording, particularly in rostral and caudal parts of CCs. In rostral parts of the brain, the CC axons turn rostrally soon after crossing the midline, and in caudal parts of the brain they turn caudally. While we were able to consistently record CAPs from mouse "whole" CCs dissected from coronal slices between positions +0.8 and -1.6, the amplitude of CAPs and, more importantly, the CAP area that reflects the number of axons, sharply dropped with distance, as many axons turned to their more proximal cortical targets or sites of origin.

"Whole" CCs from coronal slices more caudal to CC7 (bregma -1.6), while having a macroanatomical appearance of continuity across the midline, were unproductive for CAP recordings at 4 mm conduction distance symmetrically positioned around midline.

Another possible cause of rostro-caudal differences in CAP amplitudes could be the possibility of different calibers of axons, since larger axons produce larger extracellular signals. While rostro-caudal histograms of CC axon diameters have been shown in rat brain [88] (as well as in monkeys and humans [8,89,90]), the relatively small differences in axon diameters along the rostro-caudal axis in rats and lack of similar data in mice with specific reference to bregma positions do not allow at present to link possible differences in CAP amplitudes to axon diameters along the rostro-caudal axis of CCs.

An earlier study in rats on the caudal part of CC, focusing on visual neurons, used a specifically curved razor blade for cutting brain slices to follow the callosal projection of visual axons between selected contralateral visual cortical areas, allowing for successful recording of trans-callosally evoked synaptic responses in neurons [91]. No attempt was made in our study to use this approach for matching the callosal axonal projections at different rostro-caudal positions, and our slices were cut with high precision using a flat vibrating blade. Using curved razors specifically designed for different rostro-caudal parts of the brain, combined with our "whole" CC dissection, may be useful in future studies for improved recording from rostral and caudal parts of CC beyond the CC3 and CC6, with the possibility of including cortical areas for studying trans-callosally projecting cortical neurons.

### The relative amplitudes of "whole" CC CAP peaks corresponding to myelinated and non-myelinated axons

The relative sizes of peaks of ME-recorded CC CAPs vary broadly in the literature. Due to dependence of recorded signals on precise positioning of ME tip within axonal bundles, the relative sizes of N1 and N2 peaks of ME-recorded CC CAPs (“myelinated” and “non-myelinated”, respectively) vary broadly between not only different but even single publications. Our analysis of relative N1 and N2 peak amplitudes in published CC CAPs (see S1 Table) show N1>N2 (mice: [15,92,93]; rats: [10,57,58,62]), N1≈N2 (mice: [17,26,92,94]; rats: [10,13,14,16,32]), and even N1<N2 (mice: [15,17,27,29,95–98]; rats: [10,11,14,16,32,59]), which may create false impressions on the relative presence of myelinated and non-myelinated axons. This may be due to differences in precise positioning of ME tips within the CC, interference of the stimulus artifact with the fast peak of the CAP or complex interference between partially overlapping two triphasic peaks.

Our "whole" CC CAP SE recordings are devoid of above biases and invariably show much smaller higher threshold peak corresponding to non-myelinated axons (peak2), with amplitude around 25% of the faster conducting myelinated peak 1. Unlike ME recording, our SE recordings from isolated CCs are more representative of the whole populations of axons and provide reproducible ratios of myelinated and non-myelinated peaks.

The reasons for larger amplitude of peak 2 compared to peak 1 in ME-recorded CC CAPs, seen in many publications (see Introduction), remain unclear. One of the most obvious reasons could be the relatively higher number of unmyelinated axons, in 3 moth-old mice which is around 72% of CC axonal population [19]. However, because the diameters of non-myelinated axons are 2 times smaller compared to myelinated axons (mean values 0.21 μm and 0.42 μm respectively [19]), their extracellular signal should be smaller compared to that of myelinated axons. An important factor defining the size of extracellular signal may be the proximity of ME tip to the source of extracellular signal. In case of myelinated axons, the source is the nodes of Ranvier, separated by internodal distances that are in the range of 100 times the diameter of the axon, thus reducing the probability of ME tip being equally close to the nearest nodes is low. The non-myelinated axons, on the other hand, generate the signals continually along their membrane and thus may produce a larger compound extracellular signal compared to myelinated axons. Our SE recording are much less affected by variables affecting the relative size corresponding CAP peaks and provided reproducible ratios of peak2/peak1 amplitudes, which were invariably below 50% (see Fig 4B).

### Rostro-caudal patterns of conduction velocities and diameters of CC axons

Axon diameter patterns in CC vary depending on rostro-caudal position [8,88–90], suggesting differences in the speed of information exchange between corresponding cortical areas. A detailed mapping of speed of axonal conduction of action potentials (conduction velocity; CV) in different parts of CCs requires direct electrophysiological measurements. The signal conduction in myelinated and non-myelinated CC axons is distinguishable in compound action potential (CAP) recordings, as shown in many studies using ME (mice: [15,27]; rats: [10,13,14,66]; other species: [41,66]). However, no analysis of rostro-caudal patterns of CC CAP sizes and shapes had been conducted. Our present results show that conduction velocities (CVs) of myelinated axons are significantly higher in more caudal parts of CC body compared to its rostral part (see peak 1 CVs of CC6 and CC3 in Fig 4B). On the other hand, the CVs of non-myelinated axons (peak 2 CV in Fig 4B) show no statistically significant difference between CC6 and CC3.

The rostro-caudal patterns of axon diameter distribution in rat CC has been analyzed by EM and *in vivo* by a novel AxCaliber MRI methodology [88], showing broader axon diameter distribution skewed towards larger diameters in mid-CC body "segment" (corresponding to CC6 of the present study) as compared to a more rostral part of CC body corresponding to CC3 of the present study (based on the presence of anterior commissure seen in coronal brain sections). The larger axon diameters in CC6 are in good agreement with faster CVs of CC6 myelinated axons compared to CC3 found in the present study.

It should be noted that both AxCaliber and EM study of rat CC used by Barazany et al [88] did not provide data on diameters and distribution of non-myelinated axons. Such data is available from earlier EM studies of CC in rat and mice [19,66], however without analyzing the rostro-caudal patterns of axon diameters. Our data showing no statistical difference in CVs of non-myelinated axons between CC3 and CC6 suggests that there is no essential difference of the calibers of non-myelinated axons between these two CC areas.

Axons of different diameters originate from different cortical areas in macaques [9,30], suggesting a variability in conduction delays as signals converge on their targets [89,99]. Caminiti et al. [89] showed that axon diameter increased with axonal length, however this relationship was not sufficient to maintain equivalent estimated CVs across the hemisphere. While the slowest estimated CVs were found in prefrontal regions, and faster CVs in motor, somatosensory and visual areas, there was considerable variation in CVs in all areas [89]. These findings were interpreted by the authors as suggesting great variability in interhemispheric transmission speed in primates, possibly reflecting the capacity to synchronize the activity of neuron assemblies across cortical regions and between the hemispheres.

It should be noted that Caminiti et al [89] did not conduct electrophysiological measurements but rather calculated the CVs of myelinated axons from g-ratio (ratio of axon diameter to axon diameter plus myelin sheath). Because the CV also depends on other factors such as internodal distances, Na channel densities, myelin leakage and other factors that may vary between axons with different g ratios, the calculated CVs may not faithfully represent the true conduction properties of CC axons, and furthermore totally miss the non-myelinated axons. Our direct measurements of CC axonal CVs, including both myelinated and non-myelinated groups, are thus more representative of CC axonal properties, particularly due to the use of "whole" CC that more adequately represents the whole axonal populations at selected rostro-caudal planes.

### The higher complexity of axonal populations of CC

Two-peak compound action potentials (CAPs) reported in many studies using field ME recording are in agreement with ultrastructural data on the presence of myelinated and non-myelinated axons.

Here we validated the peak 1 of "whole" CC CAPs as myelinated by showing that dysmyelinated *shiverer* mice totally lack this peak but have a distinct component (Fig 5) that may reflect dysmyelinated larger diameter axons compared to non-myelinated axons. A recent study from our group using ME recording from CCs in brain slices from dysmyelinated *shiverer* mice showed only a single CAP peak, attributed to dysmyelination, and a reappearance of a two-peak CAP pattern after remyelination by transplanted stem cells [26].

In the present study, our SE recordings from “whole” CCs from *shiverer* mice revealed two distinct components, of which the first, apparently related to dysmyelinated axons that in wt would generate the myelinated peak 1, is conducted much slower than peak 1 of wt CC CAPs, while still being faster than the “non-myelinated” peak 2 of wt mice CC CAPs. Intriguingly, the “non-myelinated” peak of "whole" CC CAPs from *shiverer* mice, apparently equivalent to peak 2 of wt mice CC, is larger and faster compared to wt mice, suggesting compensatory developmental changes of axonal properties. The compensatory increase of CV may explain the data reported by Bando et al [22] who found no significant differences in trans-callosal response latencies of cortical neurons between *shiverer* (9.83 ± 0.24 ms, n = 103) and wild-type (9.33 ± 0.22 ms, n = 112) mice. Our “whole” CC CAP recordings reflect the activity of larger samples of axons compared to ME-recorded multi-unit extracellular responses in the cortex reported by Bando et al [22] and may more adequately describe the conduction properties of axons in wt and dysmyelinated mouse models.

A small 3^rd^ negative peak had been noticed in ME-recorded CAPs in rat CC slices [13] and can also be seen in some other published illustrations of ME-recorded CC CAPs in brain slices [11,14,17,59]. Our data provide strong support for the presence of a third, slower conducted peak in mouse "whole" CC CAPs. Published histograms of CC axonal calibers in ultrastructural studies [19,66,88–90,100,101] did not have sufficiently small binning that could help identify such small-diameter group of axons. Identifying the axons underlying the third CAP peak is important however is beyond the scope of this paper.

Because the SE recording is not ideally monophasic, the shape of recorded CAPs may be affected by action potential propagation inside the SE, adding a negative phase of varying size and duration to recorded CAPs. We have noticed that the negative phase of SE-recorded CAP peaks is more pronounced in slower conducted peaks, that distorts the shape of positive-going peaks. A further improvement to the methodology to selectively block action potential propagation inside the recording SE may be needed to further improve the electrophysiological analysis of CAP peaks.

Much longer conduction distances that could provide insights to the complexity of CC axonal populations had been used in ME recordings of antidromic action potentials from cortical neurons projecting through CC, evoked by stimulation of CC or the contralateral cortex *in vivo* [22,64,66]. Although these studies showed a broad range of conduction velocities (CVs), no clear peaks consistent with the two-peak CC CAPs are seen in histograms of CVs in these publications. Intracellular recording from layer V cingulate cortex neurons using *in vitro* rat brain slices [68] found CVs of antidromic action potentials ranging between 0.33 m/s and 0.8 m/s with a mean of 0.52 m/s at physiological temperature, without indication of distinct fast and slow groups. The lack of clearly defined two peaks in single-unit cortical recordings of CC-mediated antidromic responses may be due smaller number of recorded units, and larger dispersion of signal arrival times at longer distances as compared to CC recording.

Recent findings on the diversity of cortical neurons and their CC projections [4,9,102,103] suggest a greater diversity among CC axons than previously thought. However, data to support this concept are lacking. More detailed electrophysiological studies are needed to understand the complexity of CC axonal populations, beyond the well-established division into simple "myelinated" and "non-myelinated" groups. Uncovering the complexity of CC axonal populations is important both for improved understanding of normal axonal physiology and pathophysiology of CC and for the better understanding the complex interhemispheric interactions mediated by long-range and short-range CC axons. The improved accuracy of electrophysiological analysis with the novel approach presented in this paper, using suction electrode stimulation and recording from isolated CC strips from identified locations in the brain, may facilitate future studies on the possible presence of fiber subpopulations reflected in specific waveform components.

### Areas of applicability of "whole" CC electrophysiology

This study opens ways for detailed characterization of axonal conduction at various rostro-caudal CC levels of rodent brain. Although conducted in mice, this approach worked well in our preliminary experiments on rat CC. The "whole" CC can be useful for studying various experimental brain pathologies involving CC, such as myelin damage and repair, particularly in neurotrauma and MS, as well as in experimental stroke models and testing the effects of various drugs on axonal conduction in myelinated and non-myelinated axons. Because cognitive functions of the brain involve coordinated inter-hemispheric information processing, the "whole" CC electrophysiology may be of particular interest for neuroprotection studies of ageing brain in rodent models. While these areas had been productively explored with ME recording studies of CC's, the "whole" CC electrophysiology may provide more comprehensive data on the function of myelinated and non-myelinated axons in various normal and pathological conditions.

## Supporting Information

S1 Fig The arrangement of stimulating and recording suction electrodes. (PDF)

S2 Fig Comparison of electrical noise in microelectrode and suction electrode recordings. (PDF)

S3 Fig Comparison of electrical parameters of suction electrodes and microelectrodes. (PDF)

S1 Table Variability of relative peak sizes in microelectrode-recorded CC CAPs. (PDF)

S1 Video Corpus callosum dissection from a 400 μm-thick coronal slice of mouse brain. (MP4)

## Abbreviations

4-AP: 4-aminopyridine
aCSF: artificial cerebrospinal fluid
CAP: compound action potential
CC: corpus callosum
CC1…CC7: isolated CC from different rostro-caudal locations
CNS: central nervous system
CV: conduction velocity
ME: microelectrode
N1, N2: negative peaks of ME-recorded CAPs
OGD: oxygen-glucose deprivation
SE: suction electrode
shi^-/-^: *shiverer* mouse
wt: wild type mouse
