# Supplementary material for: A Novel Approach for Studying the Physiology and Pathophysiology of Myelinated and Non-Myelinated Axons in the CNS White Matter
Source: PLoS One. 2016 Nov 9;11(11):e0165637. doi: 10.1371/journal.pone.0165637 (PMC5102346; doi:10.1371/journal.pone.0165637)
Supplement: S1 Fig — (PDF) [file pone.0165637.s001.pdf]

S1 Fig

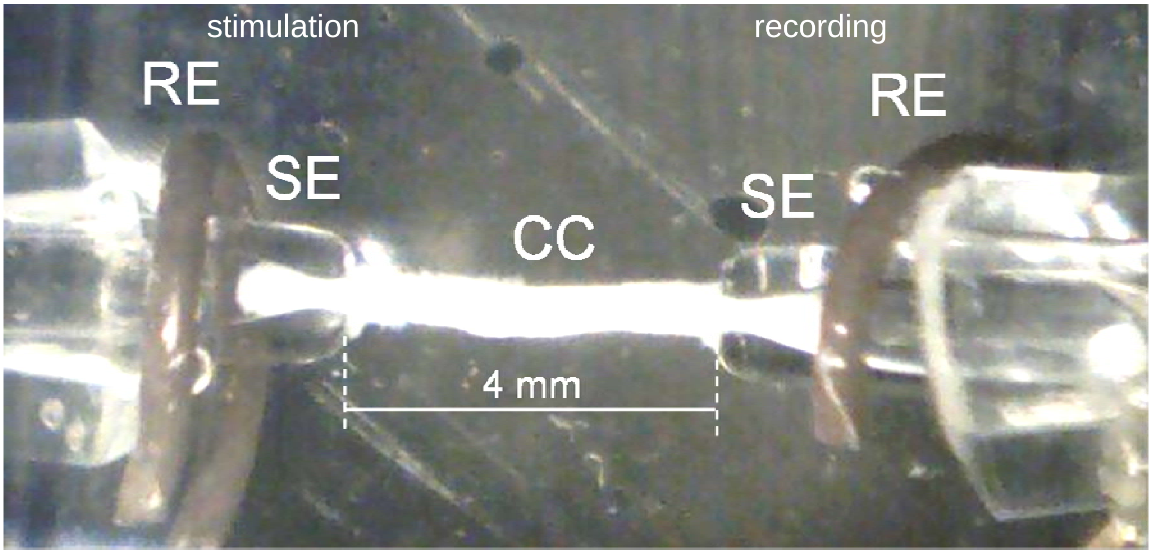

S1 Fig. The arrangement of stimulating and recording suction electrodes.
During the procedure of drawing the corpus callosum (CC) ends into suction electrodes (SEs) the remaining gray matter around the CC was stripped off, allowing for net CC recording. The reference electrodes (REs) are seen looped around the SEs, 1 mm back from their mouths. The connections of electrodes to the stimulator and the amplifier are shown in Fig 1 of the main body of the paper.
