# Supplementary material for: A Novel Approach for Studying the Physiology and Pathophysiology of Myelinated and Non-Myelinated Axons in the CNS White Matter
Source: PLoS One. 2016 Nov 9;11(11):e0165637. doi: 10.1371/journal.pone.0165637 (PMC5102346; doi:10.1371/journal.pone.0165637)
Supplement: S2 Fig — (PDF) [file pone.0165637.s002.pdf]

S2 Fig

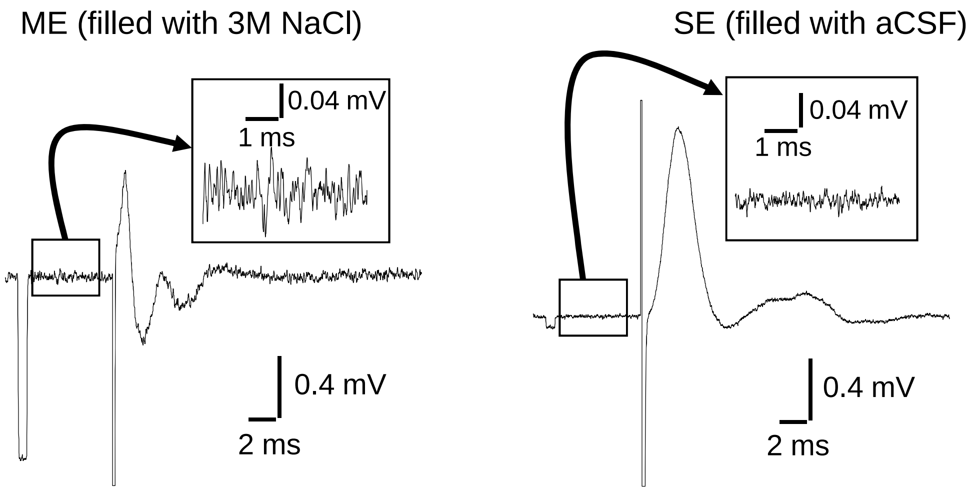

S2 Fig. Comparison of electrical noise in microelectrode and suction electrode recordings.
The negative rectangular deflections at the beginning of the traces represent responses to 1 nA * 1 ms current pulses that were used for testing the electrical resistance of electrodes. The figure shows compound action potential (CAP) recordings obtained with 3 µm-tip microelectrode (ME) and 0.3 mm i.d. suction electrode (SE). CAPs of similar peak-to-peak amplitudes of peak 1 are shown to illustrate differences in signal-to-noise between ME and SE recordings. Both CAPs are single traces and are shown at the same scale.
See S3 Fig for quantitative statistical comparisons of peak-to-peak, signal/noise and RMS noise of ME and SE recordings.
