# Supplementary material for: A Novel Approach for Studying the Physiology and Pathophysiology of Myelinated and Non-Myelinated Axons in the CNS White Matter
Source: PLoS One. 2016 Nov 9;11(11):e0165637. doi: 10.1371/journal.pone.0165637 (PMC5102346; doi:10.1371/journal.pone.0165637)
Supplement: S3 Fig — (PDF) [file pone.0165637.s003.pdf]

S3 Fig

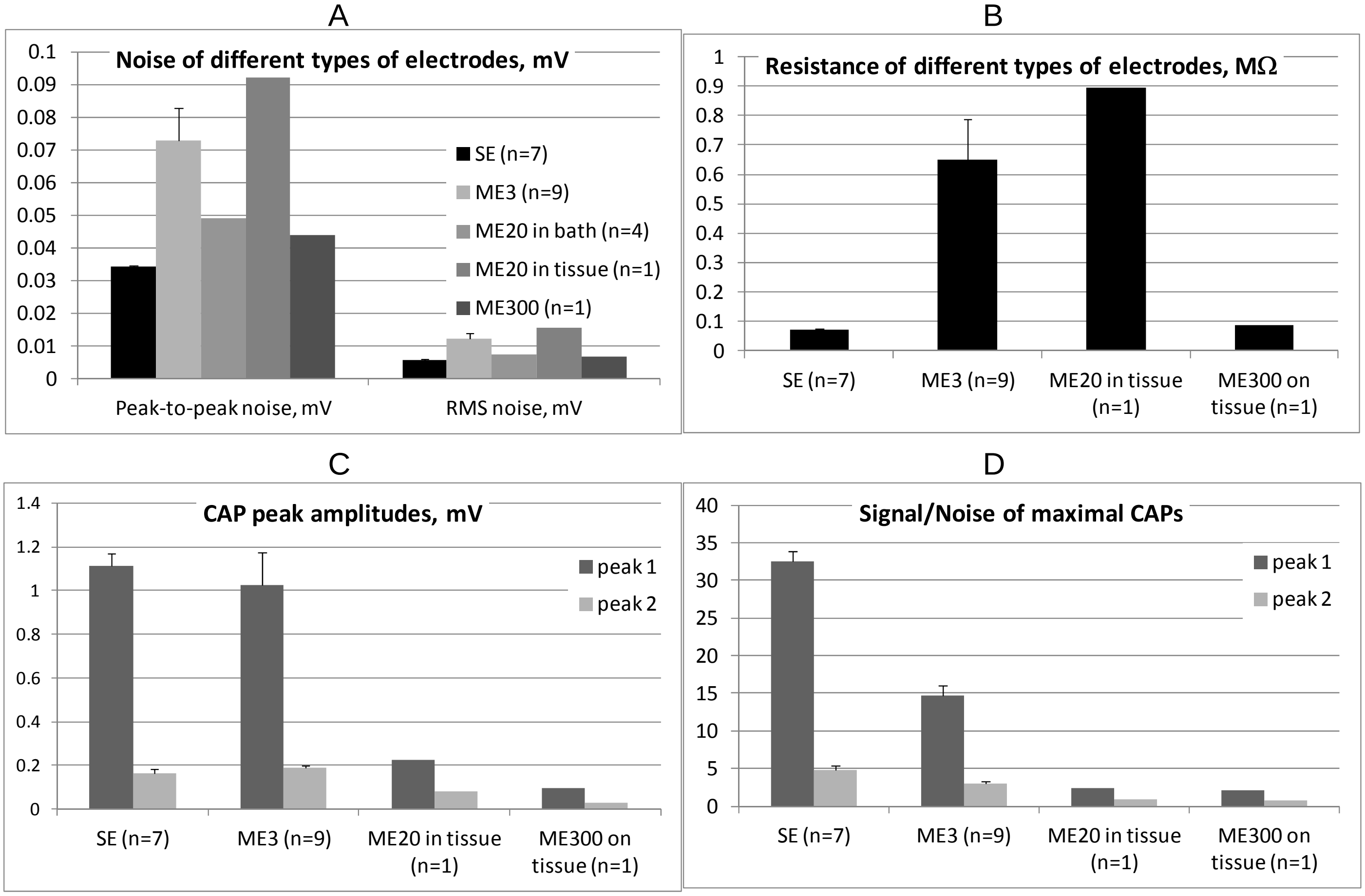

S3 Fig. Comparison of electrical parameters of suction electrodes and microelectrodes.

This figure compares the electrical noise, resistance (R) and signal/noise of suction electrodes (SE) and 2-3 µm-tip microelectrodes (ME3) used in this study, and larger diameter glass pipettes (20 µm and 300 µm; ME20 and ME300; not used in this study).

The peak–to–peak noise was measured using Statistics/Peak function of Clampfit module of pClamp10. The RMS noise was measured using Power Spectrum/Barlett function of Clampfit10. The resistance was calculated from voltage responses to 1 nA * 1 ms current pulses as described in S2 Fig.

The signal/noise was measured for maximal compound action potentials (CAPs) recorded with same electrodes shown in A-B, except for "ME20 in bath" where no CAPs recordings were performed.

SE: suction electrodes, filled with aCSF; ~300 µm "mouth" i.d., with corpus callosum (CC) inside.

ME3: filled with 3M NaCl; measurements done within CC.

ME20 and ME300: filled with ACSF. Some ME20s measurements were only done in the bath, without CAP recording. For CAP recordings, the ME3 or ME20 were inserted to a depth of 100-200 mm into the CC within the slice> When testing ME300, the tip was positioned on the surface of CC.

ME CAP recordings for this figure were performed at 1.5 mm conduction distance. CAP recordings with SE were performed at longer distances, varying between 1.8 and 4.0 mm, and still had larger amplitudes and signal/noise values compared to ME recordings.
