# Supplementary material for: A Novel Approach for Studying the Physiology and Pathophysiology of Myelinated and Non-Myelinated Axons in the CNS White Matter
Source: PLoS One. 2016 Nov 9;11(11):e0165637. doi: 10.1371/journal.pone.0165637 (PMC5102346; doi:10.1371/journal.pone.0165637)
Supplement: S1 Table — (PDF) [file pone.0165637.s004.pdf]

S1 Table. Variability of relative peak sizes in microelectrode-recorded CC CAPs.

Variability of relative sizes of two major negative peaks, N1 and N2 (myelinated and non-myelinated, respectively) in published compound action potentials (CAPs) recorded with field microelectrodes in mouse and rat corpus callosum

Species	N 1 > N 2	N1 ≈ N2	N1 < N2
Mice	Crawford et al 2009a[1]<br>Fig 1, d=2mm<br>21–23°C*****<br>ME: 3M NaCl, 1-3 MΩ	Crawford et al 2009b[2]<br>Fig 3Bi,Ci, d=2mm<br>?°C NA<br>ME: 3M NaCl, 1-3 MΩ	Baltan-Tekkok & Ransom 2004[3]<br>Fig 3**, d=2mm 33.5°C<br>ME: 2M NaCl
	Crawford et al 2009b[2]<br>Fig 2,3Ai, d=2mm<br>21–23°C<br>ME: 3M NaCl, 1-3 MΩ	Patel et al 2013[4]<br>Fig 1, d=2mm<br>21–23°C?<br>ME: 3M NaCl	Crawford et al 2009a[1]<br>Fig 2,3,4, d=2mm<br>21–23°C*****<br>ME: 3M NaCl, 1-3 MΩ
	Sahel et al 2015[5]<br>Fig 7D, d=?****<br>33°C?<br>ME: glass pipette, no details	Tekkok et al 2005[6]<br>Fig 1**, d=?<br>33°C<br>ME: 2M NaCl	Patel et al 2013[4]<br>Fig 1, d=2mm<br>21–23°C?<br>ME: 3M NaCl
		Ruff et al 2013[7]<br>Fig 5, d=?*¶¶<br>21–23°C<br>ME: 150 mM NaCl	Ritter 2012[8]<br>Fig 21, d=2mm<br>?°C NA<br>ME: ACSF, 1 MΩ
		Corcoba et al 2015 [9]<br>Fig 4, d=0.7 mm<br>28°C<br>ME: ???	Tekkok & Goldberg 2001[10]<br>Fig 2**, d=? 33°C<br>ME: 2M NaCl
			Tekkok et al 2005[11]<br>Fig 2**, d=?<br>33°C<br>ME: 2M NaCl
			Ziskin et al 2007 [12]<br>Fig 7, d=0.4..1.8mm<br>37 °C<br>ME: ACSF, 1.5-2.5 MΩ
			Olmos-Serrano et al 2016 [13]<br>Fig 6; d= varied4-point recording;<br>25°C<br>ME: ???. 1 MΩ

See next page for rat corpus callosum

Species	N 1 > N 2	N1 ≈ N2	N1 < N2
Rats	DiLeonardi et al 2012[14]<br>Fig 1,2***, d=1mm<br>?°C NA<br>ME: ACSF? 6-8 MΩ?	Baker et al 2002[15]<br>Fig 1,2, d=1 mm<br>36–37°C<br>ME: 150 mM NaCl, 2-3 MΩ	Colley et al 2010[16]<br>Fig 1,2,4,5,6, d=1mm<br>22-23°C******<br>ME: ACSF? 6-8 MΩ?
	Park et al 2011[17]<br>Fig 6, d=0.75mm<br>?°C – NA<br>ME: 150 mM NaCl, 2-3 MΩ	Colley et al 2010[16]<br>Fig 3, d=1mm<br>22-23°C******<br>ME: ACSF, 6-8 MΩ	Preston et al 1983[18]<br>Fig 1C*,3B, 4A,4C,<br>d=3.3mm **37°C - in vivo**<br>ME: 20-30 um, 0.5% agar in Ringer solution
	Preston et al 1983[18]<br>Fig 1C*, d=3.3mm<br>**37°C - in vivo**<br>ME: 20-30 um, 0.5% agar in Ringer solution	Preston et al 1983[18]<br>Fig 1C*, d=3.3mm<br>**37°C - in vivo**<br>ME: 20-30 um, 0.5% agar in Ringer solution	Reeves et al 2005[19]<br>Fig 1,2, d=1mm<br>23°C*¶<br>ME: ACSF, 6-8 MΩ
	Zhang et al 2013[20]<br>Fig 5, d=1-1.5mm<br>30°C<br>ME: 2M NaCl, 1-4 MΩ	Reeves et al 2005[19]<br>Fig 3, d=1mm<br>23°C*¶<br>ME: ACSF, 6-8 MΩ	Reeves et al 2012[21]<br>Fig 3,4, d=1.0–1.5mm<br>22–23°C<br>ME: ACSF, 2-8 MΩ
		Reeves et al 2012[21]<br>Fig 4, d=1.0–1.5mm<br>22–23°C<br>ME: ACSF, 2-8 MΩ	Schultke et al 2005[22]<br>Fig 3, d=1mm<br>37°C ("body temperature")<br>ME: 150 mM NaCl, 2-3 MΩ
			Swanson et al 1998[23]<br>Fig 2,3, d=2-3mm<br>35°C<br>ME: ACSF, 0.8-1.5 MΩ

* Depending on position of recording microelectrode - rat cc in vivo (also has an N3 peak (Preston et al 1983 - Fig 1C[18]) - conduction distance 3.3 mm.

** Peak polarities apparently reversed

*** Immature rat. Good separation from stimulus artifact, despite 1 mm distance

**** Two stimulating electrodes, monopolar tungsten; Vc = D1-D2/L

***** Discussion: "... the short latency component, N1 in the biphasic callosal CAP was obscured by the stimulus artifact when the ACSF was near physiological temperature (35–37 ◦C) as previously confirmed by Reeves et al. (2005). When the recordings were performed with the ACSF at room temperature (21–23 ◦C), conduction was slowed enough to allow separation of the N1 component from the stimulus artifact. All subsequent recordings in our lab have been performed with the ACSF at room temperature.." ..." The CAP recordings at room temperature allow the separation of stimulus artifact from the fast conducting myelinating N1 component that is lost if the recordings are performed at 35–37 ◦C (Baker et al., 2002; Reeves et al., 2005)."

****** "Electrophysiological recording was conducted with slices at room temperature (22–23 °C), based on previous findings that this range was optimal for separate quantification of the myelinated and unmyelinated CAP components (Reeves et al., 2005)".

*¶ "During initial recording sessions, it was determined that the short-latency component (N1) in the biphasic callosal CAP was obscured by the stimulus artifact, when the bath temperature was near physiological (36°C) temperature. Quantification of N1 was facilitated by lowering the bath temperature to 23°C, slowing conduction sufficiently to allow separation of this waveform component from the stimulus artifact (Fig. 1C). Accordingly, the subsequent evaluation of the effects of injury on the callosal CAPs was conducted at 23°C."

*¶¶ two-microelectrode recording for measuring conduction velocity

Reference List

1. Crawford DK, Mangiardi M, Tiwari-Woodruff SK (2009) Assaying the functional effects of demyelination and remyelination: revisiting field potential recordings. Journal of Neuroscience Methods 182: 25-33.

2. Crawford DK, Mangiardi M, Xia X, López-Valdés HE, Tiwari-Woodruff SK (2009) Functional recovery of callosal axons following demyelination: a critical window. Neuroscience 164: 1407-1421.

3. Baltan-Tekkök S, Ransom B (2004) Anoxia Effects on CNS Function and Survival: Regional Differences. Neurochemical Research 29: 2163-2169.

4. Patel R, Moore S, Crawford DK, Hannsun G, Sasidhar MV, Tan K, Molaie D, Tiwari-Woodruff SK (2013) Attenuation of Corpus Callosum Axon Myelination and Remyelination in the Absence of Circulating Sex Hormones. Brain Pathology 23: 462-475.

5. Sahel A, Ortiz FC, Kerninon C, Maldonado PP, Angulo MC, Nait-Oumesmar B (2015) Alteration of Synaptic Connectivity of Oligodendrocyte Precursor Cells Following Demyelination. Frontiers in Cellular Neuroscience 9.

6. Tekkok SB, Faddis BT, Goldberg MP (2005) AMPA/kainate receptors mediate axonal morphological disruption in hypoxic white matter. Neurosci Lett 382: 275-279.

7. Ruff CA, Ye H, Legasto JM, Stribbell NA, Wang J, Zhang L, Fehlings MG (2013) Effects of Adult Neural Precursor-Derived Myelination on Axonal Function in the Perinatal Congenitally Dysmyelinated Brain: Optimizing Time of Intervention, Developing Accurate Prediction Models, and Enhancing Performance. The Journal of Neuroscience 33: 11899-11915.

8. Ritter, Jonathan (2012) Cellular, Structural and Functional Characterization of Hyperoxia-induced White Matter Injury in the Developing Brain [dissertation]. Georgetown University.

9. Corcoba A, Steullet P, Duarte JMN, Van de Looij Y, Monin A, Cuenod M, Gruetter R, Do KQ (2016) Glutathione Deficit Affects the Integrity and Function of the Fimbria/Fornix and Anterior Commissure in Mice: Relevance for Schizophrenia. International Journal of Neuropsychopharmacology 19: 1-11.

10. Tekkok SB, Goldberg MP (2001) AMPA/kainate receptor activation mediates hypoxic oligodendrocyte death and axonal injury in cerebral white matter. J Neurosci 21: 4237-4248.

11. Tekkok SB, Faddis BT, Goldberg MP (2005) AMPA/kainate receptors mediate axonal morphological disruption in hypoxic white matter. Neurosci Lett 382: 275-279.

12. Ziskin JL, Nishiyama A, Rubio M, Fukaya M, Bergles DE (2007) Vesicular release of glutamate from unmyelinated axons in white matter. Nature Neuroscience 10: 321-330.

13. Olmos-Serrano JL, Kang HJ, Tyler WA, Silbereis JC, Cheng F, Zhu Y, Pletikos M, Jankovic-Rapan L, Cramer NP, Galdzicki Z, Goodliffe J, Peters A, Sethares C, Delalle I, Golden JA, Haydar TF, Sestan N (2016) Down Syndrome Developmental Brain Transcriptome Reveals Defective Oligodendrocyte Differentiation and Myelination. Neuron 89: 1208-1222.

14. DiLeonardi AM, Huh JW, Raghupathi R (2012) Differential Effects of FK506 on Structural and Functional Axonal Deficits After Diffuse Brain Injury in the Immature Rat. Journal of Neuropathology & Experimental Neurology 71.

15. Baker AJ, Phan N, Moulton RJ, Fehlings MG, Yucel Y, Zhao M, Liu E, Tian GF (2002) Attenuation of the electrophysiological function of the corpus callosum after fluid percussion injury in the rat. Journal of Neurotrauma 19: 587-599.

16. Colley BS, Phillips LL, Reeves TM (2010) The effects of cyclosporin-A on axonal conduction deficits following traumatic brain injury in adult rats. Experimental Neurology 224: 241-251.

17. Park E, Gottlieb JJ, Cheung B, Shek PN, Baker AJ (2011) A model of low-level primary blast brain trauma results in cytoskeletal proteolysis and chronic functional impairment in the absence of lung barotrauma. Journal of Neurotrauma 28: 343-357.

18. Preston RJ, Waxman SG, Kocsis JD (1983) Effects of 4-aminopyridine on rapidly and slowly conducting axons of rat corpus callosum. Experimental Neurology 79: 808-820.

19. Reeves TM, Phillips LL, Povlishock JT (2005) Myelinated and unmyelinated axons of the corpus callosum differ in vulnerability and functional recovery following traumatic brain injury. Experimental Neurology 196: 126-137.

20. Zhang J, Liu J, Fox HS, Xiong H (2013) N-methyl-D-aspartate receptor-mediated axonal injury in adult rat corpus callosum. J Neurosci Res 91: 240-248.

21. Reeves TM, Colley BS (2012) Electrophysiological Approaches in Traumatic Brain Injury. In: Animal Models of Acute Neurological Injuries II. pp. 313-330.

22. Schültke E, Kamencic H, Zhao M, Tian GF, Baker AJ, Griebel RW, Juurlink BH (2005) Neuroprotection following Fluid Percussion Brain Trauma: A Pilot Study Using Quercetin. Journal of Neurotrauma 22: 1475-1484.

23. Swanson TH, Krahl SE, Liu YZ, Drazba JA, Rivkees SA (1998) Evidence for physiologically active axonal adenosine receptors in the rat corpus callosum. Brain Research 784: 188-198.
